# Kommentar II zum Fall: „Eine Patientenverfügung brauche ich nicht, ich will nicht abgeschaltet werden“

**DOI:** 10.1007/s00481-022-00727-5

**Published:** 2022-11-02

**Authors:** Andreas Spickhoff

**Affiliations:** Lehrstuhl für Bürgerliches Recht und Medizinrecht, Institut für Internationales Recht, Forschungsstelle für Medizinrecht, Juristische Fakultät, LMU München, Ludwigstr. 29/I., 80539 München, Deutschland

Der Fall führt in ein ethisch wie juristisch heikles Spannungsfeld, und zwar zu der uralten Frage nach dem Rangverhältnis von Willen des Patienten auf der einen Seite und dem nach fachmedizinischen Standard Gebotenen, hier konkretisiert durch die offenbar fehlende „medizinische Indikation“ aufgrund fehlender Heilungs- und Überlebenschance.

Das Recht der Patientenverfügungen, das dem Patientenwillen besonders Rechnung tragen soll, ist in Deutschland durch eine Eigentümlichkeit gekennzeichnet: Wer sich ärztliche Maßnahmen ausdrücklich oder mutmaßlich verbittet, kann dies auch in unaufgeklärtem Zustand wirksam tun. Anders, wenn man um möglichst weitreichende ärztliche Unterstützung nachsucht: Eine solche Bitte ist als vorgezogene Einwilligung nur wirksam, wenn ihr eine korrekte ärztliche Aufklärung vorausgegangen ist. Anders gewendet wird die Bitte um umfassende ärztliche Hilfe im Vergleich zu deren unreflektierter Ablehnung erschwert. Diese tendenzielle Begünstigung der nur reduzierten ärztlichen Behandlung mag ein Hintergrund dafür sein, dass gerade ein Richter in eigener Sache Patientenverfügungen mit größter Skepsis gegenübersteht. Gleichwohl ist sein Wille prinzipiell zu akzeptieren. Hinzu getreten sein könnte die Sorge vor einer Wegnahme des lebensnotwendigen Beatmungsgerätes im Rahmen einer eventuellen „ex-post-Triage“, wenn und weil – trotz noch vorhandener Rettungsmöglichkeiten – später Patienten mit größerer Überlebenswahrscheinlichkeit konkurrierend hinzutreten. Zwar wäre eine solche ex post-Triage nach bis vor kurzem herrschender Ansicht unzulässig. Doch ist die Diskussion hierzu im Kontext der Covid 19-Pandemie in Bewegung gekommen.

Im deutschen Arztrecht ist zentraler Maßstab, nach dem Patienten zu behandeln sind, der allgemein anerkannte fachliche Standard oder Facharztstandard. Ihn definiert nach ständiger Rechtsprechung prinzipiell die (Fach‑) Ärzteschaft. Dabei ist an sich zwar auch die Festlegung einer medizinischen Indikation – von Sonderfällen wie kosmetischen Operationen abgesehen – Sache der Ärzteschaft. Indes ist die der Indikation vorgelagerte Festlegung des Behandlungsziels (an dem sich jede Indikation ausrichtet) mit dem Patienten und gemäß seinen Vorstellungen (in den Grenzen der medizinischen Möglichkeiten) festzulegen. Erst in diesem Rahmen erscheinen bestimmte fachliche Maßnahme geeignet („indiziert“), das Behandlungsziel zu erreichen (Laufs et al. 2021, Kap. VIII Rn. 95). Fehlt es an einer das Behandlungsziel und ggf. die dazu erforderlichen Maßnahmen bestimmenden wirksamen Patientenverfügung, kommt es darauf an, die Behandlungswünsche oder den mutmaßlichen Willen des Patienten festzustellen und auf dieser Grundlage zu entscheiden. Maßgeblich sind dann insbesondere frühere mündliche oder schriftliche Äußerungen, ethische oder religiöse Überzeugungen und sonstige persönliche Wertvorstellungen. Kann ein (mutmaßlicher) Wille des Patienten in die eine oder andere Richtung nicht festgestellt werden, greift als (umstrittener, aber herrschender) Grundsatz: „in dubio pro vita“.

Im vorliegenden Fall möchte der Patient eindeutig so lange wie möglich am Leben erhalten werden, in „gar keinem Fall“ toleriert er ein „Abstellen der Geräte“ im Sinne einer Therapiebegrenzung. Das Leben, auch ein qualitativ noch so schlechtes (selbst z. B. ein solches im Wachkoma), bedarf als zulässiges Behandlungsziel keiner eigenen „Indikation“ (vgl. Urteil des Bundesgerichtshofs (BGH) vom 02.04.2019 (VI ZR 13/18)^1^; Urteil des Oberlandesgerichts München vom 21.12.2017 (1 U 454/17)^2^; Spickhoff und Deuring 2019). Wohl aber ist erforderlich, dass das patientenseitige Ziel der Lebenserhaltung überhaupt noch möglich ist: Impossibilium nulla est obligatio (etwas Unmögliches wird nicht geschuldet). Im finalen Krankheitsstadium mit unzweifelhaft und unabwendbar tödlichem Ausgang (nicht vorher) wird demgemäß auch im Kontext des apallischen Syndroms eine Sondenernährung ärztlicherseits tendenziell nicht mehr empfohlen.

Wie müssen sich die behandelnden Ärzte nun im hier in Rede stehenden Fall verhalten? Müssen sie den Patienten weiterbeatmen oder können sie die Beatmung wegen fehlender Erfolgsaussicht beenden? Der Grundsatz des „in dubio pro vita“ gebietet keine Übermaßbehandlung. Vor allem kommt hier hinzu, dass der Patient einen „Automatismus in Richtung Therapiebegrenzung“ verhindern wollte. Eine Therapie seiner Erkrankung durch Weiterbeatmung ist indes weder kurativ noch sonst – von einem mehr oder weniger kurzen Aufrechterhalten der Vitalfunktionen abgesehen – überhaupt möglich. Hier liegt wohl auch ein relevanter Unterschied zur Konstellation des apallischen Syndroms, bei dem eine Besserung des Zustandes selbst in Fällen wohl nicht restlos ausgeschlossen werden kann. M. E. wäre daher die Beendigung der lebenserhaltenden Maßnahmen in der konkreten Situation rechtlich nicht zu beanstanden.

## Footnotes

1 Neue Juristische Wochenschrift 2019: 1741.

2 Zeitschrift für das gesamte Familienrecht (FamRZ) 2018: 723 als Vorinstanz (den BGH indes relativierend).
